# 4-{(4*Z*)-4-[(2*Z*)-3-(4-Fluoro­anilino)-1-hy­droxy­but-2-en-1-yl­idene]-3-methyl-5-oxo-4,5-dihydro-1*H*-pyrazol-1-yl}­benzene­sulfonamide

**DOI:** 10.1107/S1600536812006502

**Published:** 2012-02-17

**Authors:** Abdullah M. Asiri, Hassan M. Faidallah, Seik Weng Ng, Edward R. T. Tiekink

**Affiliations:** a Chemistry Department, Faculty of Science, King Abdulaziz University, PO Box 80203, Jeddah, Saudi Arabia; b The Center of Excellence for Advanced Materials Research, King Abdulaziz University, Jeddah, PO Box 80203, Saudi Arabia; c Department of Chemistry, University of Malaya, 50603 Kuala Lumpur, Malaysia

## Abstract

In the title compound, C_20_H_19_FN_4_O_4_S, the pyrazole and benzene­sulfonamide rings are coplanar [dihedral angle = 5.02 (15)°] but this planarity does not extend over the entire mol­ecule, the dihedral angle between the terminal six-membered rings being 33.24 (14)°. Intra­molecular hy­droxy–hy­droxy O—H⋯O and amine–hy­droxy N—H⋯O hydrogen bonds, as a well as a tight C—H⋯O(carbon­yl) inter­action, lead to a sequence of three fused *S*(6) rings. Supra­molecular chains along the *a* axis feature in the crystal packing; these chains are stabilized by amine–sulfonamide N—H⋯O and amine–pyrazole N—H⋯N hydrogen bonds.

## Related literature

For background to the synthesis, see: Gelin *et al.* (1983 ▶); Bendaas *et al.* (1999 ▶). For related structures, see: Asiri, Al-Youbi, Alamry *et al.* (2011 ▶); Asiri, Al-Youbi, Faidallah *et al.* (2011 ▶).
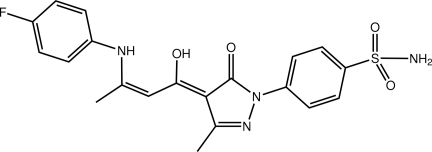


## Experimental

### 

#### Crystal data

C_20_H_19_FN_4_O_4_S
*M* *_r_* = 430.45
Triclinic, 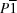

*a* = 7.8121 (5) Å
*b* = 10.0137 (8) Å
*c* = 12.6003 (9) Å
α = 97.950 (6)°
β = 104.632 (6)°
γ = 98.394 (6)°
*V* = 927.58 (12) Å^3^
*Z* = 2
Mo *K*α radiation
μ = 0.22 mm^−1^
*T* = 100 K
0.25 × 0.10 × 0.02 mm

#### Data collection

Agilent SuperNova Dual diffractometer with an Atlas detector
Absorption correction: multi-scan (*CrysAlis PRO*; Agilent, 2011 ▶) *T*_min_ = 0.946, *T*_max_ = 0.996
6559 measured reflections
4240 independent reflections
2935 reflections with *I* > 2σ(*I*)
*R*_int_ = 0.041

#### Refinement

*R*[*F* ^2^ > 2σ(*F* ^2^)] = 0.059
*wR*(*F* ^2^) = 0.146
*S* = 1.01
4240 reflections
287 parameters
4 restraints
H atoms treated by a mixture of independent and constrained refinement
Δρ_max_ = 0.47 e Å^−3^
Δρ_min_ = −0.36 e Å^−3^

### 

Data collection: *CrysAlis PRO* (Agilent, 2011 ▶); cell refinement: *CrysAlis PRO*; data reduction: *CrysAlis PRO*; program(s) used to solve structure: *SHELXS97* (Sheldrick, 2008 ▶); program(s) used to refine structure: *SHELXL97* (Sheldrick, 2008 ▶); molecular graphics: *ORTEP-3* (Farrugia, 1997 ▶) and *DIAMOND* (Brandenburg, 2006 ▶); software used to prepare material for publication: *publCIF* (Westrip, 2010 ▶).

## Supplementary Material

Crystal structure: contains datablock(s) global, I. DOI: 10.1107/S1600536812006502/hg5177sup1.cif

Structure factors: contains datablock(s) I. DOI: 10.1107/S1600536812006502/hg5177Isup2.hkl

Supplementary material file. DOI: 10.1107/S1600536812006502/hg5177Isup3.cml

Additional supplementary materials:  crystallographic information; 3D view; checkCIF report

## Figures and Tables

**Table 1:** Hydrogen-bond geometry (Å, °)

*D*—H⋯*A*	*D*—H	H⋯*A*	*D*⋯*A*	*D*—H⋯*A*
O2—H1⋯O1	0.85 (1)	1.71 (2)	2.522 (3)	161 (4)
N3—H3⋯O2	0.88 (1)	1.95 (2)	2.662 (3)	137 (3)
C2—H2⋯O1	0.95	2.26	2.917 (4)	126
N4—H41⋯N2^i^	0.88 (1)	2.14 (1)	2.999 (3)	167 (3)
N4—H42⋯O3^ii^	0.88 (1)	2.08 (1)	2.943 (3)	169 (3)

Symmetry codes: (i) 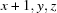; (ii) 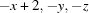.

**Fig. 1.**
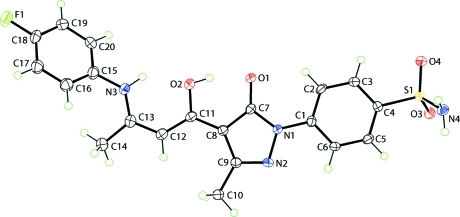
The molecular structure of (I) showing the atom-labelling scheme and displacement ellipsoids at the 50% probability level.

**Fig. 2.**
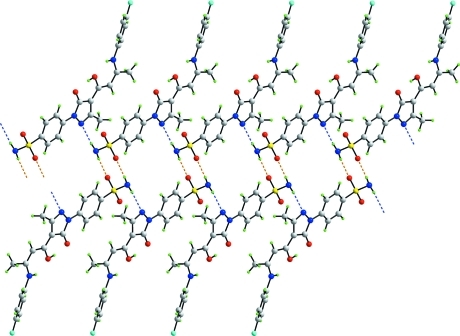
A view of a supramolecular chain along the a axis in (I) mediated by N—H···O and N—H···N hydrogen bonds shown as orange and blue dashed lines, respectively.

## supplementary crystallographic information

### Comment

In connection with a recent structural studies (Asiri, Al-Youbi, Alamry *et al.*, 2011; Asiri, Al-Youbi, Faidallah *et al.*, 2011), the title compound, 4-{4-[3-(4-fluoroanilino)-1-hydroxybut-2-enylidene]-3-methyl-5-oxo-4,5-dihydro-1*H*-pyrazol-1-yl}benzenesulfonamide (I), was prepared as a part of on-going investigations of reactions between pyrazoles and aniline derivatives based on literature procedures (Gelin *et al.*, 1983; Bendaas *et al.*, 1999).

In (I), Fig. 1, the pyrazolyl and benzenesulfonamide rings are co-planar as seen in dihedral angle of 5.02 (15)°. The planarity extends to the rest of the molecule with the exception of the terminal benzene ring; the dihedral angle between the terminal six-membered rings being 33.24 (14)°. The observed planarity is accounted for by the presence of intramolecular O—H···O and N—H···O hydrogen bonds involving the central hydroxyl-O2 atom functioning as both an acceptor and as a donor, Table 1. These interactions are complemented by a tight C—H···O1 interaction, Table 1. The intramolecular interactions lead to a sequence of three fused *S*(6) rings.

The molecules are connected into a supramolecular chain along the *a* axis in the crystal packing, Fig. 2 and Table 1. Both amide-H atoms form hydrogen bonds, one to a sulfonamide-O3 atom and the other to a pyrazolyl-N2 atom. Centrosymmetrically related molecules associate *via* an eight-membered {···HNSO}_2_ synthon. These are linked to translationally related molecules *via* N–H···N hydrogen bonds which lead to the formation of 22-membered {···HNH···OSC_4_N_2_}_2_ synthons which encompass two of the aforementioned N—H···O hydrogen bonds.

### Experimental

A solution of 4-acetoacetyl-5-hydroxy-3-methyl-1-*p*-sulfamylphenypyrazole (1.7 g, 0.005 mol) and 4-fluoroaniline (0.55 g, 0.005 mol) in ethanol (25 ml) was refluxed for 2 h. The precipitate, obtained from the hot solution, was collected, washed with methanol and recrystallized from ethanol-benzene to yield yellow crystals; *M*.pt: 411–412 K.

### Refinement

Carbon-bound H-atoms were placed in calculated positions [C—H = 0.95 to 0.98 Å, *U*_iso_(H) = 1.2 to 1.5*U*_eq_(C)] and were included in the refinement in the riding model approximation. The N—H and O—H-atoms were located in a difference Fourier map, and were refined with distance restraints of N—H = 0.88±0.01 and O—H = 0.84±0.01 Å, respectively; their *U*_iso_ values were refined.

### Crystal data

**Table:**

C_20_H_19_FN_4_O_4_S	*Z* = 2
*M**_r_* = 430.45	*F*(000) = 448
Triclinic, *P*1	*D*_x_ = 1.541 Mg m^−^^3^
Hall symbol: -P 1	Mo *K*α radiation, λ = 0.71073 Å
*a* = 7.8121 (5) Å	Cell parameters from 1846 reflections
*b* = 10.0137 (8) Å	θ = 2.4–27.5°
*c* = 12.6003 (9) Å	µ = 0.22 mm^−^^1^
α = 97.950 (6)°	*T* = 100 K
β = 104.632 (6)°	Prism, yellow
γ = 98.394 (6)°	0.25 × 0.10 × 0.02 mm
*V* = 927.58 (12) Å^3^	

### Data collection

**Table:**

Agilent SuperNova Dual diffractometer with an Atlas detector	4240 independent reflections
Radiation source: SuperNova (Mo) X-ray Source	2935 reflections with *I* > 2σ(*I*)
Mirror monochromator	*R*_int_ = 0.041
Detector resolution: 10.4041 pixels mm^-1^	θ_max_ = 27.6°, θ_min_ = 2.4°
ω scan	*h* = −10→7
Absorption correction: multi-scan (*CrysAlis PRO*; Agilent, 2011)	*k* = −12→13
*T*_min_ = 0.946, *T*_max_ = 0.996	*l* = −16→16
6559 measured reflections	

### Refinement

**Table:**

Refinement on *F*^2^	Primary atom site location: structure-invariant direct methods
Least-squares matrix: full	Secondary atom site location: difference Fourier map
*R*[*F*^2^ > 2σ(*F*^2^)] = 0.059	Hydrogen site location: inferred from neighbouring sites
*wR*(*F*^2^) = 0.146	H atoms treated by a mixture of independent and constrained refinement
*S* = 1.01	*w* = 1/[σ^2^(*F*_o_^2^) + (0.0548*P*)^2^ + 0.375*P*] where *P* = (*F*_o_^2^ + 2*F*_c_^2^)/3
4240 reflections	(Δ/σ)_max_ = 0.001
287 parameters	Δρ_max_ = 0.47 e Å^−^^3^
4 restraints	Δρ_min_ = −0.36 e Å^−^^3^

### Fractional atomic coordinates and isotropic or equivalent isotropic displacement parameters (Å^2^)

**Table:**

	*x*	*y*	*z*	*U*_iso_*/*U*_eq_	
S1	1.14758 (9)	0.18251 (7)	0.13099 (5)	0.01909 (19)	
F1	0.3342 (3)	0.78774 (18)	1.20106 (14)	0.0361 (5)	
O1	0.7345 (3)	0.41000 (19)	0.53049 (16)	0.0245 (5)	
O2	0.5322 (3)	0.4509 (2)	0.65439 (16)	0.0267 (5)	
H1	0.608 (4)	0.457 (4)	0.617 (3)	0.057 (12)*	
O3	1.0480 (3)	0.17118 (19)	0.01577 (15)	0.0218 (5)	
O4	1.2886 (3)	0.2998 (2)	0.18161 (16)	0.0262 (5)	
N1	0.6195 (3)	0.2035 (2)	0.39856 (18)	0.0181 (5)	
N2	0.4693 (3)	0.0958 (2)	0.37289 (18)	0.0194 (5)	
N3	0.3372 (3)	0.4895 (2)	0.7974 (2)	0.0246 (6)	
H3	0.429 (3)	0.519 (3)	0.772 (2)	0.026 (9)*	
N4	1.2349 (3)	0.0473 (2)	0.13729 (19)	0.0214 (5)	
H41	1.299 (3)	0.047 (3)	0.2055 (13)	0.026*	
H42	1.156 (3)	−0.025 (2)	0.098 (2)	0.026*	
C1	0.7495 (4)	0.1957 (3)	0.3386 (2)	0.0184 (6)	
C2	0.8925 (4)	0.3055 (3)	0.3569 (2)	0.0207 (6)	
H2	0.9062	0.3844	0.4119	0.025*	
C3	1.0138 (4)	0.2982 (3)	0.2943 (2)	0.0208 (6)	
H3A	1.1116	0.3725	0.3067	0.025*	
C4	0.9944 (4)	0.1833 (3)	0.2133 (2)	0.0183 (6)	
C5	0.8533 (4)	0.0721 (3)	0.1959 (2)	0.0198 (6)	
H5	0.8402	−0.0066	0.1409	0.024*	
C6	0.7327 (4)	0.0778 (3)	0.2596 (2)	0.0185 (6)	
H6	0.6385	0.0016	0.2498	0.022*	
C7	0.6167 (4)	0.3021 (3)	0.4854 (2)	0.0190 (6)	
C8	0.4559 (4)	0.2561 (3)	0.5145 (2)	0.0191 (6)	
C9	0.3752 (4)	0.1281 (3)	0.4419 (2)	0.0194 (6)	
C10	0.2070 (4)	0.0316 (3)	0.4376 (2)	0.0224 (6)	
H10A	0.1031	0.0764	0.4172	0.034*	
H10B	0.2166	0.0062	0.5110	0.034*	
H10C	0.1914	−0.0512	0.3819	0.034*	
C11	0.4130 (4)	0.3362 (3)	0.6027 (2)	0.0215 (6)	
C12	0.2592 (4)	0.3066 (3)	0.6396 (2)	0.0252 (7)	
H12	0.1707	0.2292	0.5978	0.030*	
C13	0.2232 (4)	0.3787 (3)	0.7311 (2)	0.0233 (6)	
C14	0.0456 (4)	0.3322 (3)	0.7541 (3)	0.0316 (8)	
H14A	−0.0467	0.2940	0.6834	0.047*	
H14B	0.0101	0.4107	0.7929	0.047*	
H14C	0.0581	0.2617	0.8008	0.047*	
C15	0.3261 (4)	0.5620 (3)	0.9014 (2)	0.0273 (7)	
C16	0.2655 (4)	0.4976 (3)	0.9798 (3)	0.0288 (7)	
H16	0.2238	0.4011	0.9648	0.035*	
C17	0.2665 (4)	0.5744 (3)	1.0786 (2)	0.0281 (7)	
H17	0.2213	0.5319	1.1315	0.034*	
C18	0.3323 (4)	0.7119 (3)	1.1012 (2)	0.0252 (7)	
C19	0.3989 (4)	0.7777 (3)	1.0272 (2)	0.0280 (7)	
H19	0.4477	0.8733	1.0453	0.034*	
C20	0.3932 (4)	0.7016 (3)	0.9259 (2)	0.0257 (7)	
H20	0.4356	0.7454	0.8727	0.031*	

### Atomic displacement parameters (Å^2^)

**Table:**

	*U*^11^	*U*^22^	*U*^33^	*U*^12^	*U*^13^	*U*^23^
S1	0.0177 (4)	0.0186 (4)	0.0193 (4)	−0.0007 (3)	0.0069 (3)	−0.0013 (3)
F1	0.0487 (12)	0.0327 (10)	0.0289 (10)	0.0100 (9)	0.0165 (9)	−0.0006 (8)
O1	0.0214 (11)	0.0215 (10)	0.0259 (11)	−0.0032 (9)	0.0089 (9)	−0.0082 (8)
O2	0.0252 (12)	0.0268 (11)	0.0250 (11)	−0.0012 (10)	0.0107 (9)	−0.0059 (9)
O3	0.0224 (10)	0.0235 (11)	0.0189 (10)	0.0035 (9)	0.0055 (8)	0.0029 (8)
O4	0.0244 (11)	0.0233 (11)	0.0281 (11)	−0.0044 (9)	0.0119 (9)	−0.0040 (8)
N1	0.0169 (12)	0.0174 (12)	0.0177 (12)	0.0004 (10)	0.0053 (9)	−0.0020 (9)
N2	0.0171 (12)	0.0186 (12)	0.0197 (12)	−0.0013 (10)	0.0044 (10)	0.0000 (9)
N3	0.0236 (14)	0.0240 (13)	0.0243 (13)	0.0026 (11)	0.0092 (11)	−0.0042 (10)
N4	0.0205 (13)	0.0233 (13)	0.0170 (12)	0.0033 (11)	0.0022 (10)	−0.0009 (10)
C1	0.0181 (14)	0.0220 (14)	0.0156 (13)	0.0053 (12)	0.0053 (11)	0.0018 (11)
C2	0.0246 (15)	0.0169 (14)	0.0172 (14)	0.0033 (12)	0.0045 (12)	−0.0047 (10)
C3	0.0188 (14)	0.0181 (14)	0.0231 (15)	0.0003 (12)	0.0052 (12)	−0.0001 (11)
C4	0.0160 (14)	0.0200 (14)	0.0192 (14)	0.0029 (12)	0.0063 (11)	0.0023 (11)
C5	0.0208 (15)	0.0174 (14)	0.0182 (14)	0.0027 (12)	0.0030 (11)	−0.0018 (10)
C6	0.0164 (14)	0.0174 (14)	0.0185 (14)	−0.0013 (11)	0.0036 (11)	0.0000 (10)
C7	0.0213 (15)	0.0195 (14)	0.0146 (13)	0.0029 (12)	0.0042 (11)	−0.0002 (10)
C8	0.0177 (14)	0.0209 (14)	0.0175 (14)	0.0028 (12)	0.0044 (11)	0.0010 (11)
C9	0.0179 (14)	0.0203 (14)	0.0186 (14)	0.0023 (12)	0.0039 (11)	0.0019 (11)
C10	0.0239 (15)	0.0200 (14)	0.0214 (15)	−0.0005 (12)	0.0070 (12)	0.0012 (11)
C11	0.0226 (15)	0.0211 (14)	0.0176 (14)	0.0021 (12)	0.0036 (12)	−0.0008 (11)
C12	0.0251 (16)	0.0254 (16)	0.0222 (15)	0.0021 (13)	0.0059 (12)	−0.0011 (12)
C13	0.0245 (15)	0.0252 (15)	0.0215 (15)	0.0072 (13)	0.0074 (12)	0.0041 (12)
C14	0.0278 (17)	0.0327 (18)	0.0347 (18)	0.0034 (15)	0.0146 (14)	−0.0008 (13)
C15	0.0244 (16)	0.0315 (17)	0.0254 (16)	0.0072 (14)	0.0083 (13)	−0.0006 (12)
C16	0.0314 (17)	0.0256 (16)	0.0308 (17)	0.0067 (14)	0.0105 (14)	0.0048 (13)
C17	0.0290 (17)	0.0287 (17)	0.0284 (17)	0.0074 (14)	0.0110 (14)	0.0037 (13)
C18	0.0260 (16)	0.0307 (16)	0.0207 (15)	0.0123 (14)	0.0077 (12)	0.0014 (12)
C19	0.0263 (16)	0.0247 (16)	0.0302 (17)	0.0003 (14)	0.0090 (13)	−0.0016 (12)
C20	0.0203 (15)	0.0287 (16)	0.0285 (16)	0.0021 (13)	0.0096 (13)	0.0039 (13)

### Geometric parameters (Å, º)

**Table:**

S1—O4	1.4353 (19)	C6—H6	0.9500
S1—O3	1.4437 (19)	C7—C8	1.427 (4)
S1—N4	1.605 (3)	C8—C11	1.417 (4)
S1—C4	1.770 (3)	C8—C9	1.423 (4)
F1—C18	1.372 (3)	C9—C10	1.497 (4)
O1—C7	1.270 (3)	C10—H10A	0.9800
O2—C11	1.336 (3)	C10—H10B	0.9800
O2—H1	0.848 (10)	C10—H10C	0.9800
N1—C7	1.375 (3)	C11—C12	1.402 (4)
N1—N2	1.410 (3)	C12—C13	1.387 (4)
N1—C1	1.415 (3)	C12—H12	0.9500
N2—C9	1.311 (4)	C13—C14	1.508 (4)
N3—C13	1.342 (4)	C14—H14A	0.9800
N3—C15	1.436 (4)	C14—H14B	0.9800
N3—H3	0.881 (10)	C14—H14C	0.9800
N4—H41	0.878 (10)	C15—C20	1.382 (4)
N4—H42	0.875 (10)	C15—C16	1.393 (4)
C1—C2	1.397 (4)	C16—C17	1.368 (4)
C1—C6	1.401 (4)	C16—H16	0.9500
C2—C3	1.381 (4)	C17—C18	1.361 (4)
C2—H2	0.9500	C17—H17	0.9500
C3—C4	1.391 (4)	C18—C19	1.375 (4)
C3—H3A	0.9500	C19—C20	1.380 (4)
C4—C5	1.398 (4)	C19—H19	0.9500
C5—C6	1.386 (4)	C20—H20	0.9500
C5—H5	0.9500		
			
O4—S1—O3	118.53 (11)	N2—C9—C10	119.3 (2)
O4—S1—N4	108.00 (13)	C8—C9—C10	128.9 (3)
O3—S1—N4	106.03 (12)	C9—C10—H10A	109.5
O4—S1—C4	106.19 (12)	C9—C10—H10B	109.5
O3—S1—C4	108.51 (13)	H10A—C10—H10B	109.5
N4—S1—C4	109.38 (13)	C9—C10—H10C	109.5
C11—O2—H1	105 (3)	H10A—C10—H10C	109.5
C7—N1—N2	111.1 (2)	H10B—C10—H10C	109.5
C7—N1—C1	129.7 (2)	O2—C11—C12	117.8 (3)
N2—N1—C1	119.2 (2)	O2—C11—C8	115.8 (2)
C9—N2—N1	106.2 (2)	C12—C11—C8	126.4 (2)
C13—N3—C15	127.8 (2)	C13—C12—C11	126.6 (3)
C13—N3—H3	114 (2)	C13—C12—H12	116.7
C15—N3—H3	118 (2)	C11—C12—H12	116.7
S1—N4—H41	111 (2)	N3—C13—C12	122.7 (3)
S1—N4—H42	110 (2)	N3—C13—C14	119.2 (3)
H41—N4—H42	121 (3)	C12—C13—C14	118.1 (3)
C2—C1—C6	120.2 (2)	C13—C14—H14A	109.5
C2—C1—N1	120.2 (2)	C13—C14—H14B	109.5
C6—C1—N1	119.6 (2)	H14A—C14—H14B	109.5
C3—C2—C1	119.3 (3)	C13—C14—H14C	109.5
C3—C2—H2	120.3	H14A—C14—H14C	109.5
C1—C2—H2	120.3	H14B—C14—H14C	109.5
C2—C3—C4	120.7 (2)	C20—C15—C16	119.7 (3)
C2—C3—H3A	119.6	C20—C15—N3	116.5 (2)
C4—C3—H3A	119.6	C16—C15—N3	123.6 (3)
C3—C4—C5	120.2 (3)	C17—C16—C15	119.5 (3)
C3—C4—S1	118.9 (2)	C17—C16—H16	120.2
C5—C4—S1	120.9 (2)	C15—C16—H16	120.2
C6—C5—C4	119.4 (2)	C18—C17—C16	119.9 (3)
C6—C5—H5	120.3	C18—C17—H17	120.0
C4—C5—H5	120.3	C16—C17—H17	120.0
C5—C6—C1	120.1 (2)	C17—C18—F1	119.4 (2)
C5—C6—H6	120.0	C17—C18—C19	122.0 (3)
C1—C6—H6	120.0	F1—C18—C19	118.6 (3)
O1—C7—N1	126.8 (2)	C18—C19—C20	118.4 (3)
O1—C7—C8	127.7 (2)	C18—C19—H19	120.8
N1—C7—C8	105.5 (2)	C20—C19—H19	120.8
C11—C8—C9	134.9 (3)	C19—C20—C15	120.4 (3)
C11—C8—C7	119.6 (2)	C19—C20—H20	119.8
C9—C8—C7	105.5 (2)	C15—C20—H20	119.8
N2—C9—C8	111.8 (2)		
			
C7—N1—N2—C9	0.5 (3)	N1—C7—C8—C9	1.2 (3)
C1—N1—N2—C9	178.6 (2)	N1—N2—C9—C8	0.3 (3)
C7—N1—C1—C2	−7.0 (4)	N1—N2—C9—C10	−178.6 (2)
N2—N1—C1—C2	175.4 (2)	C11—C8—C9—N2	−179.4 (3)
C7—N1—C1—C6	173.8 (3)	C7—C8—C9—N2	−1.0 (3)
N2—N1—C1—C6	−3.8 (4)	C11—C8—C9—C10	−0.6 (6)
C6—C1—C2—C3	1.8 (4)	C7—C8—C9—C10	177.8 (3)
N1—C1—C2—C3	−177.4 (3)	C9—C8—C11—O2	177.0 (3)
C1—C2—C3—C4	0.3 (4)	C7—C8—C11—O2	−1.2 (4)
C2—C3—C4—C5	−1.4 (4)	C9—C8—C11—C12	−3.1 (6)
C2—C3—C4—S1	177.1 (2)	C7—C8—C11—C12	178.6 (3)
O4—S1—C4—C3	10.3 (3)	O2—C11—C12—C13	−5.5 (5)
O3—S1—C4—C3	−118.1 (2)	C8—C11—C12—C13	174.7 (3)
N4—S1—C4—C3	126.6 (2)	C15—N3—C13—C12	−171.6 (3)
O4—S1—C4—C5	−171.2 (2)	C15—N3—C13—C14	10.4 (5)
O3—S1—C4—C5	60.3 (3)	C11—C12—C13—N3	−0.2 (5)
N4—S1—C4—C5	−54.9 (3)	C11—C12—C13—C14	177.9 (3)
C3—C4—C5—C6	0.3 (4)	C13—N3—C15—C20	−148.1 (3)
S1—C4—C5—C6	−178.1 (2)	C13—N3—C15—C16	37.0 (5)
C4—C5—C6—C1	1.8 (4)	C20—C15—C16—C17	2.6 (5)
C2—C1—C6—C5	−2.9 (4)	N3—C15—C16—C17	177.3 (3)
N1—C1—C6—C5	176.3 (3)	C15—C16—C17—C18	−2.3 (5)
N2—N1—C7—O1	178.0 (3)	C16—C17—C18—F1	−179.4 (3)
C1—N1—C7—O1	0.2 (5)	C16—C17—C18—C19	0.0 (5)
N2—N1—C7—C8	−1.1 (3)	C17—C18—C19—C20	1.9 (5)
C1—N1—C7—C8	−178.9 (3)	F1—C18—C19—C20	−178.6 (3)
O1—C7—C8—C11	0.9 (5)	C18—C19—C20—C15	−1.6 (5)
N1—C7—C8—C11	179.9 (2)	C16—C15—C20—C19	−0.6 (5)
O1—C7—C8—C9	−177.8 (3)	N3—C15—C20—C19	−175.7 (3)

### Hydrogen-bond geometry (Å, º)

**Table:**

*D*—H···*A*	*D*—H	H···*A*	*D*···*A*	*D*—H···*A*
O2—H1···O1	0.85 (1)	1.71 (2)	2.522 (3)	161 (4)
N3—H3···O2	0.88 (1)	1.95 (2)	2.662 (3)	137 (3)
C2—H2···O1	0.95	2.26	2.917 (4)	126
N4—H41···N2^i^	0.88 (1)	2.14 (1)	2.999 (3)	167 (3)
N4—H42···O3^ii^	0.88 (1)	2.08 (1)	2.943 (3)	169 (3)

Symmetry codes: (i) *x*+1, *y*, *z*; (ii) −*x*+2, −*y*, −*z*.
